# Psychological Distress and Work Environment Perception by Physical Therapists from Southern Italy during COVID-19 Pandemic: The C.A.L.A.B.R.I.A Study

**DOI:** 10.3390/ijerph18189676

**Published:** 2021-09-14

**Authors:** Alessandro de Sire, Nicola Marotta, Simona Raimo, Lorenzo Lippi, Maria Teresa Inzitari, Anna Tasselli, Alessandra Gimigliano, Liana Palermo, Marco Invernizzi, Antonio Ammendolia

**Affiliations:** 1 Physical Medicine and Rehabilitation Unit, Department of Medical and Surgical Sciences, University of Catanzaro “Magna Graecia”, 88100 Catanzaro, Italy; inzitari@unicz.it (M.T.I.); annatasselli@gmail.com (A.T.); ammendolia@unicz.it (A.A.); 2 Laboratory of Cognitive Psychology, Department of Medical and Surgical Sciences, University of Catanzaro “Magna Graecia”, 88100 Catanzaro, Italy; simona.raimo@unicz.it (S.R.); liana.palermo@unicz.it (L.P.); 3 Physical and Rehabilitative Medicine, Department of Health Sciences, University of Eastern Piedmont, 28100 Novara, Italy; lorenzolippi.mt@gmail.com (L.L.); marco.invernizzi@med.uniupo.it (M.I.); 4 Chief Medical Officer, ASST Fatebenefratelli-Sacco, 20157 Milan, Italy; alessandra.gimigliano@gmail.com; 5 Translational Medicine, Dipartimento Attività Integrate Ricerca e Innovazione (DAIRI), Azienda Ospedaliera SS. Antonio e Biagio e Cesare Arrigo, 15121 Alessandria, Italy

**Keywords:** COVID-19, psychology, physical therapy, rehabilitation, work environment, healthcare, burnout, depression, healthcare workers

## Abstract

The psychosocial impact of the work environment during the COVID-19 pandemic on health professionals is a growing issue. The present study examined specific psychosocial work environment indicators during the COVID-19 pandemic, through a multiple regression model of a self-administered cross-sectional online survey in a cohort of physical therapists from a region of Southern Italy from March 2020 to May 2021. The questionnaire contained items on work and healthcare issues related to COVID-19. Eighty physical therapists (29 male and 51 female), mean age 32.5 ± 10.1 years, were involved in this survey. The multiple regression analysis showed that “management activity” was significantly correlated to “therapist frustration” during the COVID-19 pandemic (ΔR^2^ = 0.16; *p* < 0.03). Findings of this study underline the importance of a healthy psychosocial work environment to enhance job satisfaction of all health professionals and to avoid role conflict and burnout syndrome during the COVID-19 pandemic.

## 1. Introduction

The outbreak of severe acute respiratory syndrome coronavirus 2 (SARS-CoV-2), which started in late December 2019 in Hubei Province in China, caused millions of cases of coronavirus disease (COVID-19) worldwide in just a few months and evolved into a pandemic [1]. In Italy, from 3 January 2020 to 11 June 2021, there have been 4,239,868 confirmed cases of COVID-19 with 126,855 deaths, as reported by the World Health Organization [2]. 

Overall, in October 2020, the research on this topic progressively shifted from the description of COVID-19 related disability in the acute phase (almost half of papers published in 2020) to complications in the sub-acute (33.9%) and chronic phase (4.8%). In early 2021, the research shed light on putative predictive factors for unfavorable outcomes and severe consequences of COVID-19 infection on long-lasting activity limitations and participation restrictions [2]. To date, large numbers of COVID-19 patients require outpatient and home rehabilitation care and the negative impact of the pandemic on Rehabilitation Units’ performance and organization still causes deficiencies in meeting patient needs [3]. In this scenario, the remodeling of hospitals and territorial rehabilitation services would still be necessary, while recommendations on telerehabilitation approaches still do not seem to be established [4].

Early rehabilitation treatment begins immediately after discharge from intensive care and clinically stable COVID-19 patients in the post-acute phase have undergone a rehabilitation protocol aimed at reducing dyspnea and improving gas exchange, muscle function and autonomy in activities of daily living (ADL) [5].

The dramatic spread of the current COVID-19 pandemic in Italy has spurred the establishment of multidisciplinary operators to address the complex and multifaceted disabling sequelae affecting these patients and to set up comprehensive and effective rehabilitative interventions. The so-called “respiratory rehabilitators” (pulmonologists and physical therapists), who have been involved for years in the treatment of patients with disabilities secondary to pathological respiratory conditions, are the health professionals most involved in this complex rehabilitative framework. Their experience gained in the management of chronic and acute respiratory failure is proving to be a fundamental reference point for the management of patients needing rehabilitative interventions during the COVID-19 epidemic, although the disabling sequelae of COVID-19 are not only related to the respiratory system but also include neuromuscular and cognitive impairments [6,7,8]. Hence, the reorganization involved in taking care of this scenario is not likely to be a short-term issue [9]. In short, previous research has provided insight into the relationships between personality, age, gender, coping styles, social support, and emotional distress during the COVID-19 pandemic [10,11]. 

A recent study performed in 12 different countries found that the COVID-19 pandemic disrupted the usual rehabilitation services, with the negative consequence that many patients are not receiving the correct rehabilitative care. In this context, infection control procedures posed significant barriers to providing effective rehabilitative interventions [12]. Moreover, hospitals have also requested greater support from physical and rehabilitative medicine (PRM) physicians due to the high amount of COVID-19 patient admittances. Thus, their usual clinical activities have been dramatically reduced. Moreover, the decisions of the Italian government aimed at slowing down the pandemic phenomenon and reduce the burden on the emergency departments reduced outpatient access and hospital admissions for chronic patients [13]. Taken together, all these issues have strongly limited access to treatment for rehabilitative patients.

Koffman et al. reported that uncertainty is pervasive in healthcare with overlapping sources including ethical and systems uncertainty for health systems, providers, patients, and families in addressing doubts and concerns during the COVID19 epidemic. In this context, it is recommended to set up new management workflows tailored for the pandemic, rehearsing and exploring imaginative ways to show empathy and collaborate with patients/families in order to assist physical therapists in coping with a “new normal” [14,15].

Shechter et al. suggested that severe COVID-19-related psychological distress is expected to have downstream impacts on healthcare workers’ physical health, in light of the well-known association between clinical workplace environmental stressors and long -term cardiometabolic risk, exerted by both direct (systemic inflammation, arterial damage, increased blood pressure) and indirect (maladaptive coping such as substance use, poor sleep) factors [16]. In this context, considering the basic psychological needs in terms of frustration, rather than a lack of satisfaction, it is crucial to provide a more adequate understanding of the damaging effects that psychological distress might have in the workplace [17].

As a whole, learning of new protocols and procedures at work, dealing with an exceedingly large number of patients and sustaining long shifts with protective equipment along with the fear of getting infected, has led healthcare workers to perceive a lower quality work environment relationships with colleagues [18]. This has often resulted in developing emotional and behavioral symptoms such as excessive worries, mood swings, sleep difficulties and eating disorders [19,20]. Given this background, key protective factors have been identified to help healthcare workers to cope with the work situation in a more resilient way, limiting distress [21]. These latter have included both individual characteristics (e.g., resilient coping style and self-esteem) [22], and perceived characteristics of the work environment (e.g., sense of work group cohesion and coherence) that have been shown to be protective measures against distress [23,24].

In this scenario, a better understanding of the relationships between clinical and emotional outcomes would increase our knowledge of these protective or vulnerability factors associated with the development of negative emotions in healthcare workers [25]. Moreover, in addition to the personal experiences of trauma, several reports have underlined many operational difficulties stemming from power hierarchies, inequality and a perceived disconnection between senior management and front-line staff [26,27].

Therefore, the aim of the present study is to assess the correlation between work environmental factors and psychological distress in a cohort of physical therapists working in hospitals in Southern Italy during the COVID-19 pandemic.

## 2. Materials and Methods

### 2.1. Participants

In this local cross-sectional survey, entitled “COVID-19: A Long And Brief Rehabilitative Interdisciplinary Approach (C.A.L.A.B.R.I.A) study”, we enrolled a cohort of Italian physical therapists involved in the management of patients with post-acute COVID-19 in Calabria, a Region of Southern Italy. All study participants were recruited from March 2020 to May 2021 through an online recruitment strategy and were asked to fill in a questionnaire, after a detailed description of the survey and previous authorization by the participants to be contacted for survey purposes. 

Inclusion criteria were: (a) physical therapists working in COVID-19 clinics; (b) physical therapists involved in the management of patients with COVID-19; (c) consent to share their data with the researchers (which guaranteed privacy protection and permission for distribution of the survey). The exclusion criteria were previous diagnosis of psychiatric disorder or a history of confirmed infection with COVID-19. This study was approved by the Institutional Review Board and was performed in accordance with pertinent national regulatory requirements. All the participants were asked to carefully read and sign an informed consent before collecting the data and privacy protection was guaranteed by the study investigators. 

### 2.2. Survey Questionnaire 

All participants were asked to complete a nine-item questionnaire built for the C.A.L.A.B.R.I.A study to evaluate the correlation among work environmental determinants and psychological distress in a cohort of physical therapists in hospitals in Southern Italy during the second wave of the COVID-19 pandemic. The survey questionnaire was created by a group of technical experts, composed of 6 PRM physicians, 2 cognitive psychologists, 1 physical therapist, and 1 healthcare director. This investigation was in agreement with previous studies already published in the literature focusing on occupational environmental factors [28]. 

The questionnaire started with a brief description (including aim) of the C.A.L.A.B.R.I.A study, also reporting the online informed consent agreement via the following statement: “I have read the consent and agree to participate”. We performed an online recruitment strategy through E-mails, Facebook, and WhatsApp, to administer the link to the present survey. 

Before starting the online survey, all study participants were asked to provide information on personal data (e.g., age, gender, work experience, etc.). The questionnaire consisted of two different domains: domain A) consisted of open-ended questions describing epidemiologic and job information during the COVID-19 pandemic; domain B) consisted of questions on psychological distress and work environmental factors, assessed by a 7-point Likert scale [29] (ranging from 1 = never to 7 = always). The questionnaire is shown in detail in Table 1.

### 2.3. Statistical Analysis

The statistical analysis was performed using JASP 0.14 (JASP Team, Amsterdam, The Netherlands). Data are presented as means ± standard deviations (SD) for continuous variables, and counts and percentages for dichotomous, nominal, and ordinal variables. Kendall’s correlations τ were conducted to investigate the extent of association between two sets of ranked order data, with range of values from −1 to 1 [30,31]. In more detail, we performed a sequential multiple regression considering Q7 (“How much do you feel frustrated in relation to your work?”) as criterion value (CV) and inserting a fixed number of predictive variables (PV) (Q4, Q5, Q6, Q8, and Q9). Hence, the objective of this sequential multiple regression is to estimate the individual contribution of each PV in changing the CV (Q7) variance, reported as change in R^2^ (denoted as delta R^2^ or ΔR^2^) [32]. ΔR^2^ resulted from entering each PV into the above-mentioned regression mode, emerging from entering each PV into a regression design [32], and consists of physical therapists’ frustration change according to the other variables [32]. Moreover, we also estimated the Root Mean Square Error (RMSE) as the standard deviation of the residuals (prediction errors) to enrich the quality of the model. Residuals are considered as a distance measure from data points of the regression line; thus, the lower the RMSE, the better the model [33]. Finally, we performed the F-statistic giving the overall significance of the model, evaluating if at least one predictor variable has a non-zero coefficient [34].

## 3. Results

**Figure 1:**
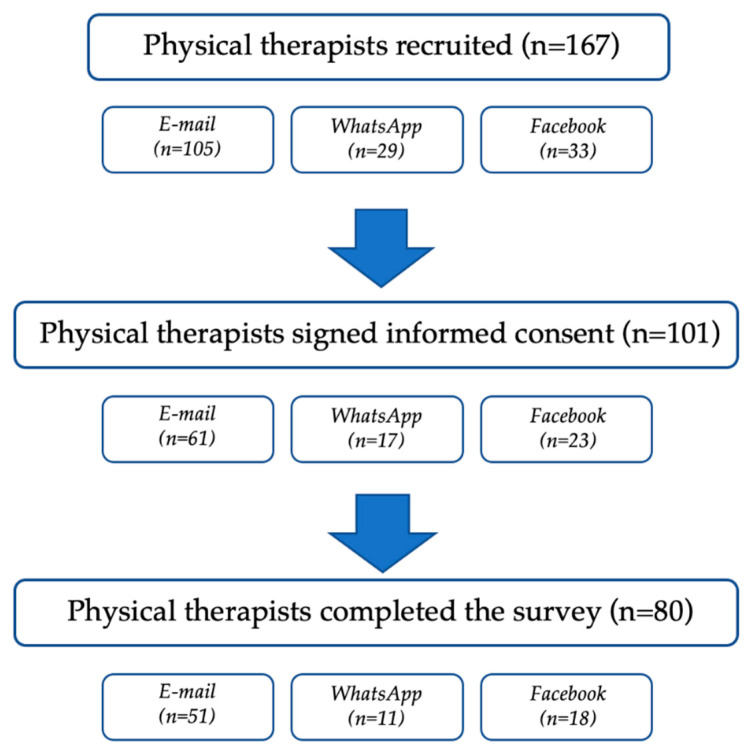
Survey response flow chart.

Out of 167 physical therapists recruited through an online recruitment strategy, 101 physical therapists adequately signed the informed consent. However, 18 forgot to answer at least one psychological impact-related question, and three did not send the form, thus being excluded from the analysis (see Figure 1 for further details).

Therefore, 80 physical therapists (29 male and 51 female), mean aged 32.5 ± 10.1 years, were involved in this survey, as depicted in Table 2.

Regarding the first question (Q1), 42 (52.5%) participants had worked in the Calabria Region for less than 5 years, 23 (28.8%) for more than 5 years, and 21 (26.2%) for less than a year. Forty-nine (61.3%) physical therapists were hired by private facilities affiliated with the Italian National Health System, while 31 (38.7%) were hired by the NHS. Regarding question Q2, 64 (80.0%), participants stated that their job duties had totally changed, 14 (17.5%) considered their job had partially changed, while only 2 (2.5%) replied that their job duties had not changed at all. Regarding question Q3, 75% of participants revealed that they had not followed an adequate training course for the rehabilitation of patients with COVID-19.

Concerning the questions on psychological distress and work environmental factors, the physical therapists involved in the study reported the following mean values using the 7-point Likert scale:

- Question 4 (Q4—“How much do you feel sure of your working skills during COVID-19 pandemic?”) = 4.3 ± 1.4;
- Question 5 (Q5—“How much does your superior actively work to ensure an optimal organization of the activities?”) = 4.6 ± 1.2;
- Question 6 (Q6—“How much does your superior actively work to ensure good conditions of well-being and employee development?”) = 4.5 ± 1.2;
- Question 7 (Q7—“How much do you feel frustrated in relation to your work?”) = 3.9 ± 1.2;
- Question 8 (Q8—“How much do you feel exposed to the risk of being affected by COVID-19”) = 4.8 ± 1.3;
- Question 9 (Q9—“How much do you fear your colleagues being affected by COVID-19?”) = 4.9 ± 1.1.

As shown in Table 3, we performed Kendall’s τ correlation based on the working sector (private = 0; public = 1) and the gender (male = 0; female = 1), obtaining a significant negative Kendall’s τ correlation between the question of the public or private nature of the physiotherapist’s work with a τ = −0.32; (*p* < 0.01) for Q4, and a τ = −0.48 (*p* < 0.001) for Q6. The physiotherapists that work in the public sector reported a higher confidence in their skills and their direct superiors worked more to ensure good conditions of well-being.

Furthermore, a significant positive Kendall’s τ correlation was found between Q4 and Q5 (τ = 0.52; *p* < 0.001); τ = 0.36 in Q4:Q6 (*p* = 0.03); τ = 0.50 in Q5:Q6 (*p* < 0.001), τ = 0.39 in Q5:Q7 (*p* = 0.03); τ = 0.50 in Q7:Q9 (*p* < 0.004); τ = 0.69 in Q8:Q9 (*p* < 0.001) (see Table 4 for further details).

**Figure 2:**
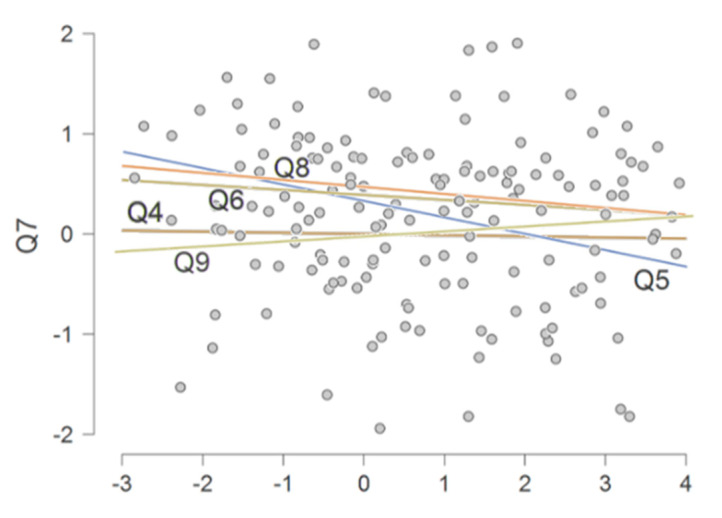
Multiple regression scatterplot, estimating the individual contribution of each predictive variable (Q4, Q5, Q6, Q8, and Q9) in changing the variance of the criterion value (Q7: “To what extent did you feel frustrated in relation to your work?”), to estimate the individual contribution of each answer by physical therapists to the other specific questions on psychological distress and work environmental factors.

Moreover, by performing a sequential multiple regression, we evaluated each question in relation to the variation made to the frustration perceived by each participant (Q7) (see Figure 2). 

Therefore, after the multiple regression analysis, we reported a significant ΔR^2^ of 0.16 (*p* = 0.03) of Q5 regarding Q7, with a RMSE = 1.12.

## 4. Discussion

Other recent studies have showed a high prevalence of rehabilitative healthcare professionals’ burnout during the COVID-19 pandemic [35,36]. However, to the best of our knowledge, this is the first study investigating the impact that the psychosocial work environment might have on frustration perceived by physical therapists. 

Indeed, this cross-sectional survey interestingly highlighted that the 16% incremental variance of the frustration related to the work of the physical therapists involved might be explained by the question “How much does your superior actively work to ensure an optimal organization of the activities? (Q5)”. More in detail, the variation in the therapist’s frustration depended on activity by their superiors in ensuring optimal working conditions during the COVID-19 pandemic. 

Recommendations have been addressed to support the implementation of a multidisciplinary rehabilitative pathway for those COVID-19 patients in need of functional recovery [37]. In this context, early post-acute rehabilitation including mobilization and respiratory physiotherapy measures may have a significant impact on patient recovery after COVID-19 and may be especially important for patients who needed critical care medicine with or without mechanical ventilation and are at risk for the development of post-intensive care syndrome [38]. Recent studies reported that a significant percentage of healthcare workers, including physical therapists, suffer from psychological problems such as depression, insomnia, obsessive-compulsive symptoms and anxiety related to the workplace and general issues related to the COVID-19 pandemic [39,40]. Indeed, manual and direct contact with patients increases fear and worries of contracting and transmitting the virus to family members, leading to the promotion of partially effective treatment strategies including not touching the patient unless necessary, necessitating patients to use face masks, excessive use of sanitizers and the provision of instructions to patients from a distance [41]. Thus, considering that healthcare workers are vulnerable to psychological symptoms, and that such symptoms lasting for long periods lead to allostatic overload [42], the mental health of physical therapists should be constantly and cautiously monitored [39]. 

The reorganization of the rehabilitation services exploited the professional and clinical skills of the physiotherapists [43]. It is well known that physiotherapists consider clinical supervision to be effective, especially if it favors the development of their professional skills. Indeed, the physical therapists preferred a direct supervision model, in which their supervisor directly observed and guided the development of their professional expertise [44]. Additionally, physical therapists recognized the importance of informal supervision in which guidance is provided from supervisors who appreciate the supervision process [45].

The analysis of the demographic data correlations showed two significant associations between the private or public employment relationship and these two questions: “Q4—How much do you feel sure of your working skills during COVID-19 pandemic?” and “Q6—How much does your superior actively work to ensure good conditions of well-being and employee development?”. In more detail, the relationship between private and public employment and self-reported working skills might be partly related to the different reallocation rates in different units. On the other hand, a recent study [46] underlined that one out five physiotherapists were reallocated in different wards (such as infective wards, pulmonology wards, or intensive care units) during COVID-19 pandemic as a result of clinical PRM departments’ reorganization. Therefore, changes in tasks might significantly affect the operator’s perceived job skills.

Similarly, the positive relation between direct superior care of workers’ well-being and work setting might be strictly linked to the difference in infection risk in physiotherapists called to face the COVID-19 pandemic. It might not be surprising to understand how anxiety and depression might be associated with direct superior commitment to ensure good working conditions. The suggestions and ideas proposed by frontline healthcare workers contribute to the maintenance of appropriate equipment and optimal hygiene protocols in high-risk departments such as intensive care units [47], and generate a feeling of security and motivation that boost the work efficiency and motivation of all the team [48,49]. Therefore, these data highlighted the need for further research focusing on the type of work environment and not just the physiotherapist’s relationship with the work environment [50]. Moreover, the multiple regression showed a consistent correlation of frustration with all of the domains analyzed. However, a total of 16% of the variation in frustration could be explained by the active work of the superior perceived by the therapist. Beyond the degree of raw association of the single predictive values obtained through a sequential multiple regression analysis, we are able to weigh how much a predictor explains a variation of a dependent criterion. In this scenario, the need for continuous improvement of qualifications is essential for the medical professions, and the perception of the usefulness and effectiveness of one’s work is one of the main factors in combating burnout [36]. 

Physical therapists and all health care workers are among individuals with the highest risk of contracting the virus, considering that they are under an overwhelming psychological pressure, requiring manual contact with patients [35,41,43,48]. It has been proposed that focusing on lack of basic need satisfaction alone may be ineffective in adequately describing how negative work-related social factors impact employees’ psychological needs [17,41]. Indeed, many operational difficulties stemmed from inequalities of role between management and front-line workers. The work frustration by healthcare workers might be more clinically relevant than their work satisfaction in explaining how the COVID-19 pandemic has negatively affected the rehabilitation field. We are aware that learning from this experience could reduce staff distress and improve patient care in case of further waves of this pandemic. 

Thus, in the context of the ongoing pandemic, social support could be dominantly viewed as a useful option for an individual to rely on and use as emotional support to cope with traumatic stress [3,4]. Evidence shows that social support is associated with resilience to stress and the reduction of depression and anxiety. Therefore, social support could play a key role in maintaining mental and physical health, not only in health workers but also in the general population [51]. 

We are aware that the present study is not free from limitations. First, a multiple regression approach would require a larger sample, but a low RMSE ensured the validity of the analysis. Second, the study was unable to distinguish pre-existing mental health symptoms from new symptoms, albeit the different sense of frustration felt in the various waves remains difficult to resolve. Third, it would have been useful to use widespread, standardized, and validated scales, despite that a panel of experts had formulated the online questionnaire that should be reliable for all physiotherapists involved in counteracting this COVID-19 pandemic. Thus, future research is needed to address the above limitations, including the comparison of the presence of anxiety and depression between physical therapists and a control group.

## 5. Conclusions

Taken together, findings of the C.A.L.A.B.R.I.A. suggest the need to constantly monitor the needs of rehabilitative healthcare workers and to implement tailored psychological intervention programs during the COVID-19 pandemic. In more detail, the variation in the therapist’s frustration seems to be strictly related to the role of medical directors in ensuring a safe environment to reduce burnout in healthcare worker in the rehabilitation field.

## Figures and Tables

**Table 1:** Online questionnaire aimed at evaluating epidemiological data, psychological distress and work environmental factors in physical therapists involved in the C.A.L.A.B.R.I.A. study.

Domain A: Epidemiologic questions
(Q1) How long have you been working in this Region and in which sector do you work?
(Q2) What has significantly changed in your work duties during COVID-19 pandemic?
(Q3) Have you performed a specific course/training to manage COVID-19 issues in your workplace?
Domain B: Questions on psychological distress and work environment (7-point Likert scale)
(Q4) How much do you feel sure of your working skills during COVID-19 pandemic?
(Q5) How much does your superior actively work to ensure an optimal organization of the activities?
(Q6) How much does your superior actively work to ensure good conditions of well-being and employee development?
(Q7) How much do you feel frustrated in relation to your work?
(Q8) How much do you feel exposed to the risk of being affected by COVID-19?
(Q9) How much do you fear your colleagues being affected by COVID-19?

**Table 2:** Demographic characteristics of participants.

	Total (*n* = 80)
Male/female	29/51
Age (years)	32.5 ± 10.1
Physical therapists working in the private sector (%)	49 (61.3)
Physical therapists working in the National Health System (%)	31 (38.7)

Data are presented as mean and standard deviation for continuous variables; counts and percentages for categorical variables; x/y for ratios.

**Table 3:** Kendall’s τ correlations concerning demographical data of physical therapists involved in the C.A.L.A.B.R.I.A. study (*n* = 80).

Variable	In Which Sector Do You Work?	Age	Gender
In which sector do you work?	—		
Age	0.04	—	
Gender	0.14	0.03	—
Q4	−0.32 *	0.13	−0.04
Q5	−0.02	−0.06	−0.06
Q6	−0.48 **	0.01	−0.16
Q7	0.16	0.18	−0.09
Q8	0.06	−0.03	0.09
Q9	0.16	0.08	0.05

* = *p* < 0.01, ** = *p* < 0.001.

**Table 4:** Kendall’s τ correlations concerning the answers to the questions on psychological distress and work environmental factors by physical therapists involved in the C.A.L.A.B.R.I.A. study (*n* = 80).

Variable	Q4	Q5	Q6	Q7	Q8	Q9
1. Q4	—					
2. Q5	0.52 ***	—				
3. Q6	0.36 *	0.50 ***	—			
4. Q7	−0.24	−0.30 *	−0.27	—		
5. Q8	−0.03	−0.26	0.01	0.31	—	
6. Q9	−0.04	−0.06	−0.24 *	0.50 **	0.69 ***	—

* = *p* < 0.05; ** = *p* <0.01; *** = *p* < 0.001.

## Data Availability

Not applicable.
